# Optimal Response to Quorum-Sensing Signals Varies in Different Host Environments with Different Pathogen Group Size

**DOI:** 10.1128/mBio.00535-20

**Published:** 2020-06-02

**Authors:** Liqin Zhou, Leyla Slamti, Didier Lereclus, Ben Raymond

**Affiliations:** a School of Biological Sciences, Royal Holloway University of London, Egham, United Kingdom; b Université Paris-Saclay, INRAE, AgroParisTech, Micalis Institute, Jouy-en-Josas, France; c Imperial College London, Ascot, United Kingdom; d University of Exeter, Penryn, United Kingdom; University of Pittsburgh

**Keywords:** cheating, cooperation, PlcR/PapR, polymorphism, signaling, virulence regulation

## Abstract

The persistence of genetic variation in master regulators of gene expression, such as quorum-sensing systems, is hard to explain. Here, we investigated two alternative hypotheses for the prevalence of polymorphic quorum sensing in Gram-positive bacteria, i.e., the use of different signal/receptor pairs (‘pherotypes’) to regulate the same functions. First, social interactions between pherotypes or ‘facultative cheating’ may favor rare variants that exploit the signals of others. Second, different pherotypes may increase fitness in different environments. We evaluated these hypotheses in the invertebrate pathogen Bacillus thuringiensis, using three pherotypes expressed in a common genetic background. Facultative cheating could occur in well-mixed host homogenates provided there was minimal cross talk between competing pherotypes. However, facultative cheating did not occur when spatial structure was increased in static cultures or in naturalistic oral infections, where common pherotypes had higher fitness. There was clear support for environment-dependent fitness; pherotypes varied in responsiveness to signals and in mean competitive fitness. Notably, competitive fitness varied with group size. In contrast to typical social evolution models of quorum sensing which predict higher response to signal at larger group size, the pherotype with highest responsiveness to signals performed best in smaller hosts where infections have a lower pathogen group size. In this system, low signal abundance appears to limit fitness in hosts, while the optimal level of response to signals varies in different host environments.

## Abstract (precis)

Quorum sensing describes the ability of microbes to alter gene regulation according to their local population size. Some successful theory suggests that this is a form of cooperation, namely, investment in shared products is only worthwhile if there are sufficient bacteria making the same product. This theory can explain the genetic diversity in these signaling systems in Gram-positive bacteria, such as *Bacillus* and *Staphylococcus* sp. The possible advantages gained by rare genotypes (which can exploit the products of their more common neighbors) could explain why different genotypes can coexist. We show that while these social interactions can occur in simple laboratory experiments, they do not occur in naturalistic infections using an invertebrate pathogen, Bacillus thuringiensis. Instead, our results suggest that different genotypes are adapted to differently sized hosts. Overall, social models are not easily applied to this system, implying that a different explanation for this form of quorum sensing is required.

## INTRODUCTION

Pathogenic bacteria often coordinate the secretion of a wide range of virulence factors via ‘quorum sensing’ (QS), the release of, and response to, small diffusible autoinducer molecules (1, 2). Since the concentration of autoinducers in the environment increases with cell density, these molecules can drive density and growth phase-dependent changes in gene expression (1, 2). The evolutionary biology of quorum sensing has been closely linked to theories of cooperation. QS signaling molecules can behave as ‘public goods’ (3, 4), products, or services that benefit entire communities, but require costly contributions from individuals. In addition, QS systems may regulate the expression of virulence factors that are also public goods (3, 4). Autoinducer molecules, especially oligopeptides, are metabolically costly (5). Nonproducers can behave as ‘cheats’; in mixed culture, they gain a growth advantage by avoiding costs of signaling and exploiting the signals produced by others (3, 4, 6–8). Importantly, if QS regulates the production of typical public goods, theory predicts increased levels of public goods should increase group level benefits, typically total bacterial population size (4, 9–11). However, in pathogens such as Bacillus thuringiensis, virulence factors do not behave as classical public goods and confer benefits via increased mortality or invasiveness (12, 13).

QS systems are diverse both within and between species. Gram-negative bacteria often have systems based on acyl-homoserine lactones, while Gram-positive bacteria commonly have peptide-based autoinducing signals (2, 14). Many species have multiple QS systems with different mechanisms activating different genes (15). However, in many peptide systems in members of *Bacillus*, *Streptococcus*, and *Staphylococcus*, there is unexplained genetic diversity in QS signals and receptors that activate the same presumptive suite of genes (16–20). Different QS alleles can be found within closely related clades, and multiple alleles may co-occur at a fine spatial scale (21–23). The functional significance of these polymorphic signal variants, known as pherotypes, is largely unknown.

**FIG 1:**
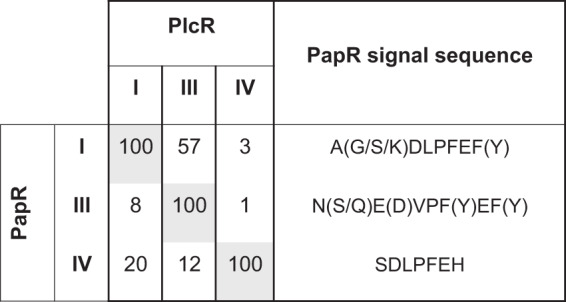
Variation in responsiveness and in cross talk between QS pherotypes in the B. cereus group. Each column presents the % of transcriptional activity of P*_plcA_*, a PlcR-dependent promoter, when the indicated peptide is added to the cells relative to its activity when the cognate peptide is used for each PlcR pherotype. Each column should be read independently of the others. PlcR I, III, and IV indicate the *plcR* allele harbored by an otherwise isogenic *ΔplcRpapR* strain. PapR I, III, and IV indicate the pherotype of the synthetic peptide added to the cells. The figure was produced using data from Bouillaut et al. (23).

In the Bacillus cereus group, PlcR is a master regulator of virulence; it controls the production of a large number of proteases and phospholipases with putative roles in host cell lysis, in addition to proteins involved in suppressing host immunity and bacterial competition (24, 25). The cellulolytic function of many of these enzymes explains why PlcR substantially increases the efficacy of host invasion from the gut during oral infection (12, 26). At least one gene in the PlcR regulon (*inhA2*) is an immune suppressor (27). However, B. thuringiensis uses diverse strategies to be a potent suppressor of immune function in insects, and most of these do not depend on PlcR (28). PlcR is activated by the binding of the signaling peptide PapR (29). There are four distinct pherotypes (I, II, III, and IV) in the PlcR/PapR system (20, 23). Each pherotype (hereafter, pp I to IV) corresponds to a distinct signaling peptide (Fig. 1), but these pherotype alleles are not restricted to particular clades or species (20). For instance, all four pherotype alleles have been found in B. thuringiensis (20), a specialized invertebrate pathogen widely distributed in the environment (30). There are various degrees of ‘cross talk’ between pherotypes in this system, with this being the activation of one receptor by the signal from another pherotype. Receptor-peptide interactions can be highly specific in pp IV, for example, while pp I, II, and III receptors can be activated to a lesser extent by peptides from other pherotypes (23) (Fig. 1). PlcR receptor proteins vary in the region involved in signal binding but have highly conserved helix-turn-helix domains, the region involved in DNA binding (23, 31); different PlcR pherotypes are expected to activate a similar suite of genes. Thus, the persistence of diverse pherotypes remains a puzzle.

One evolutionary phenomenon that can maintain high levels of polymorphism is negative frequency dependence, the concept that rare variants can have a fitness advantage over more common types. Social interactions (cooperation/cheating) between QS variants can provide a mechanism for negative frequency dependence; QS cheaters or nonproducers commonly have higher fitness when rare in mixed cultures (3, 4, 6–8). One form of social exploitation that could drive the maintenance of polymorphism is facultative cheating (8, 32, 33). Facultative cheating can occur if pherotypes respond less efficiently to signals from bacteria with different QS alleles, i.e., if cross talk between variants is limited. Rare pherotypes may act as transient cheats since, at low frequencies, they will have low-level responses to their own QS signals but may still exploit the secreted products of the common pherotypes. Facultative cheating can drive frequency-dependent cheating behavior and negative frequency-dependent fitness (8, 32).

An alternative, but not mutually exclusive, hypothesis for QS polymorphism is that different pherotypes have various fitness in distinct environments (16, 34). Importantly, in the B. cereus group, putative hosts encompass vertebrates, insects, and nematodes, with body sizes ranging over several orders of magnitude; while not all species in the group are specialized as pathogens, some are opportunists or colonizers of cadavers (35, 36). Different QS pherotypes vary in the degree of responsiveness to their own signal peptides; standard concentrations of signal produce different levels of transcription of a PlcR-dependent reporter (23). The structural basis of the interactions between PapR and PlcR leading to the activation of PlcR has been reported for pp I (31, 37), and structural modeling has provided insight into the binding of PapR II, III, or IV to their corresponding PlcR variant (23). Overall, more responsive pherotypes are expected to invest more in virulence factors than less responsive counterparts. One important environmental variable predicting investment in QS-dependent virulence is group size; increased investment in cooperative virulence should be favored in larger groups (9), while group size, in turn, may be linked to the size of hosts.

We tested hypotheses experimentally, using different pherotypes from diverse B. thuringiensis strains expressed in a common genetic background. Our key hypotheses make distinct predictions. The facultative cheating hypothesis predicts that alleles will have higher fitness when rare in mixed infections (negative frequency dependence) and assumes that all QS is phenotypically equivalent, while the environment-dependent hypothesis predicts that distinct alleles produce distinct phenotypes which vary in fitness according to environmental conditions. In addition, we tested the environment-dependent fitness hypothesis by characterizing the phenotypic differences conferred by different pherotype alleles. Since early results indicated that the relative fitness of competing pherotypes varied with abundance of B. thuringiensis in oral infections, we also tested how factors that determine pathogen group size (i.e., size of host and pathogen dose) affect competitive fitness.

Existing support for facultative cheating comes from simple *in vitro* systems which use relatively homogeneous, well-mixed conditions on artificial media where coexisting genotypes can exploit each other’s secreted products (8, 32, 38). We investigated whether facultative cheating is also theoretically possible in the B. cereus group by conducting experiments in the well-mixed environment provided by cultures in insect homogenates. We also explored the consequence of increasing spatial structure via the use of static cultures on the fitness of potential cheats, with our expectation being that spatial structure should reduce cheater fitness (39–41). Finally, we tested hypotheses in ecologically realistic oral infections, the typical mode of infection for B. thuringiensis (42). An important feature of the B. thuringiensis system is that insect hosts are a complex and realistic experimental model in which physical structure, population bottlenecks, and separation of coinfecting genotypes can restrict cheating (12, 43).

## RESULTS

### Differences in expression of QS-regulated virulence factors between pherotypes.

**FIG 2:**
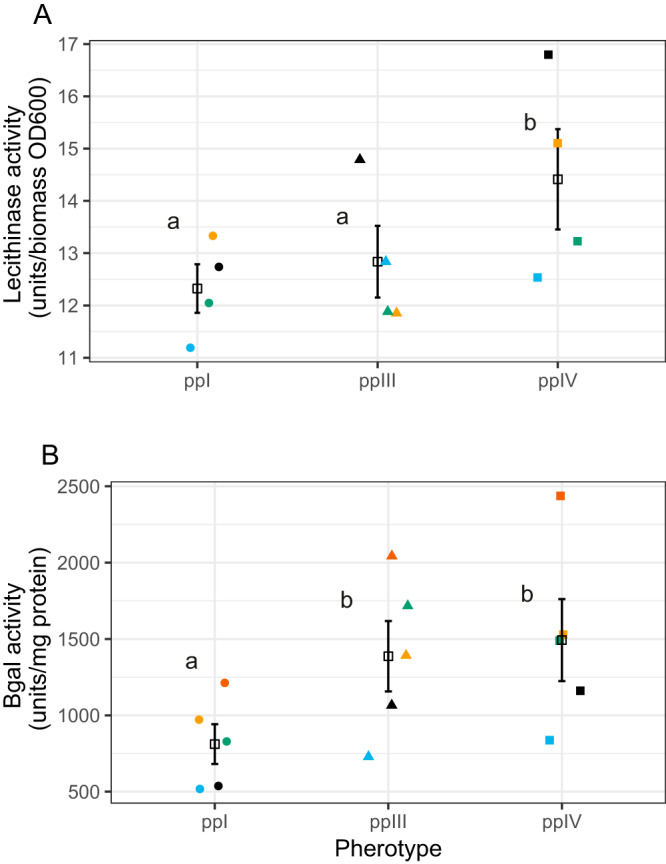
Variation in PlcR activity in near-isogenic mutants with different pherotypes. This activity was determined in two different independent assays by measuring the activity of a PlcR/PapR-regulated gene product or of a PlcR/PapR-dependent promoter. (A) Peak lecithinase activity square root-transformed, and (B) peak β-galactosidase activity. Data are independent repeats, color coded by block; open squares and error bars are means ± SE. Different letters indicate significant differences in *post hoc* treatment contrasts. In this and all subsequent figures, ppI, ppIII, and ppIV indicate the pherotype I, III, and IV constructs expressed in a common genetic background.

The experiment used three pherotype groups described in B. cereus. The chosen groups were marked to enable pairwise competition, in which there were various levels of cross talk; we expected minimal cross talk between pp III and IV but more cross talk between pp I and IV (Fig. 1). In order to determine if there was a variation in PlcR activity between pherotypes, we measured the production of QS-regulated lecithinase *in vitro* via hydrolysis of egg yolk phosphatidylcholine. We also conducted assays of QS-dependent transcription rate, measured by a β-galactosidase reporter. Peak (maximal) enzyme activity for lecithinase and β-galactosidase occurred 1 and 2 hours after entry into the transition phase between exponential growth and stationary phase and was consistent across all pherotypes (data not shown). Pherotype IV had the highest peak of lecithinase activity (Fig. 2A) (square root-transformed data; mixed model likelihood ratio test, 7.04; degrees of freedom [df] = 2; *P* = 0.0295), while the other two pherotypes were indistinguishable from each other (likelihood ratio test, 0.61; df = 1; *P* = 0.44). Although there were subtle differences between the lecithinase and β-galactosidase assays, there was variation between pherotypes in both experiments (Fig. 2B) (likelihood ratio test, 16.4; df = 2; *P* = 0.0003). Pherotype IV had the highest β-galactosidase activity in 4/5 experiments, but activity in pp IV and pp III was not significantly different (likelihood ratio test, 0.83; df = 1; *P* = 0.36), while both pp III and pp IV pherotypes had higher levels of β-galactosidase activity than the pp I strain (*post hoc t* = 5.69, *P* = 0.0003).

### Facultative cheating and productivity in homogenized host environments.

**FIG 3:**
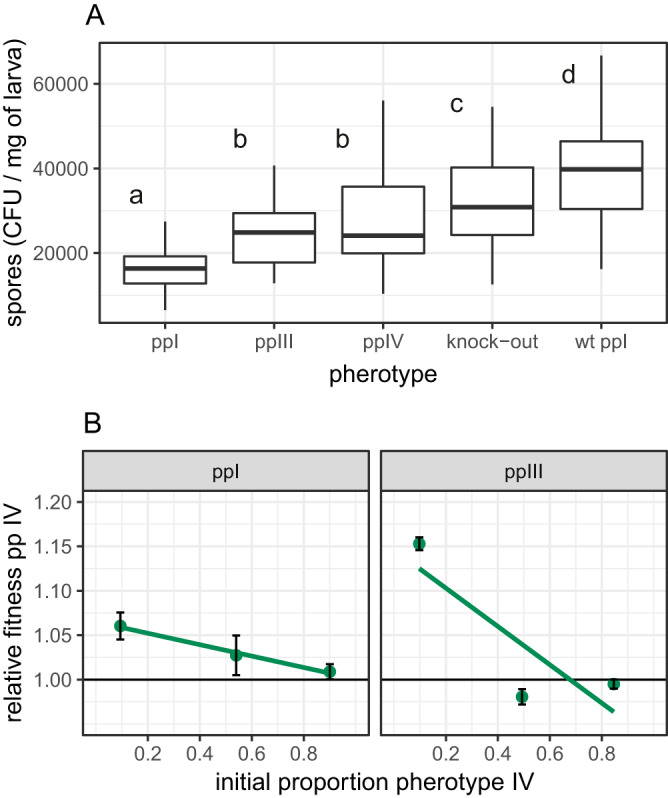
Variation in total reproduction and of the relative fitness of B. thuringiensis pherotypes in homogenized insects. (A) Variation in final population size in numbers of spores per unit weight of insects between three pherotypes; boxplots with different letters indicate pherotypes that are significantly different in *post hoc* treatment contrasts; wt ppI indicates strain B. thuringiensis 407 Cry^−^ expressing chromosomally encoded PlcR-PapR belonging to pp I. Boxplots are drawn with default ggplot2 settings and show the median and the first and third quartiles, with the whiskers extending to values no further than 1.5× the interquartile range. (B) Variation in fitness with frequency of competitors in two sets of experiments with different pherotype pairs; data are means ± SE with fitted models for each competition treatment.

Here, we tested the main prediction of the facultative cheating hypothesis that pherotypes should have a fitness advantage when rare. Based on our previous work with null mutants, we further predicted that negative frequency-dependent fitness would be more likely to occur in well-mixed environments (insect homogenates) and that expression of PapR and PlcR would provide benefits in terms of increased population size (final production of spores) in insect homogenates. As predicted, there was significant variation in population size between treatment groups (Fig. 3A) (generalized linear model [GLM], *F*_4,158_ = 30.5, *P* < 0.0001). The wild-type (WT) ppI strain also produced significantly more spores than the double-knockout Δ*plcR-papR* (test by model simplification, *F*_1,160_ = 6.56, *P = *0.011).

We also expected that the more responsive pherotypes might have increased group-level benefits and, therefore, produce more spores in insect homogenates. Thus, while pp IV and pp III have a similar final population size (test by pooling of treatment levels, *F*_1,158_ = 0.36, *P = *0.55), both of these pherotypes were more productive than the less responsive pp I (Fig. 3A) (*t* = 5.48, *P* < 0.0001). There was, however, a clear cost in reduced population size in the artificial plasmid construct of pp I relative to the WT (Fig. 3A) (*t* = 10.4, *P* < 0.0001).

As predicted by the facultative cheating hypothesis, pherotype fitness increased when rare in homogenized insect media (Fig. 3B) (pp IV/III competition, *F*_1,70_ = 104, *P < *0.0001; pp IV/I competition, *F*_1,69_ = 5.04, *P = *0.028). Fitness patterns were not symmetrical, however. Pherotype IV mutants, when rare, could clearly outcompete pp III since, at 10% frequency, fitness was greater than 1 (Fig. 3B) (mean fitness, 1.153; 95% confidence intervals, 1.139 to 1.167). However, rare pp III mutants could not outcompete pp IV (Fig. 3B) (mean fitness, 0.995; 95% confidence intervals, 0.98 to 1.01). Overall, the change in fitness with frequency was greatest in competitions between pp IV and III (GLM, pherotype × frequency interaction, *F*_1,139_ = 17.7, *P < *0.0001).

### Effect of varied spatial structure in static and shaken insect homogenates.

**FIG 4:**
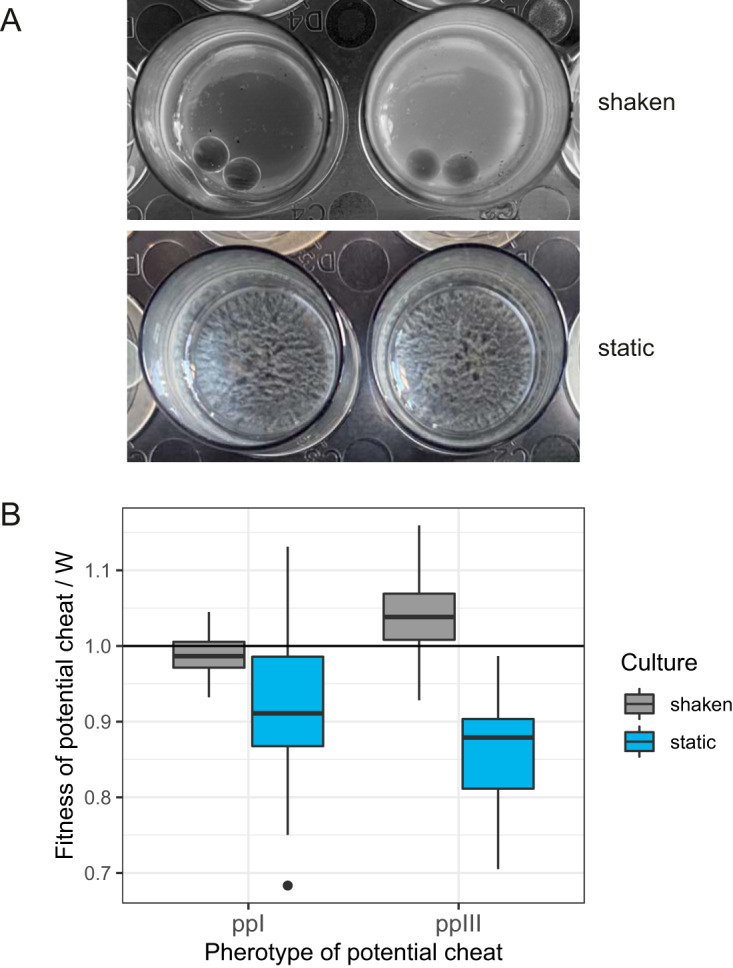
The impact of varying spatial structure in static and shaken insect homogenates on the fitness of rare pherotypes in competition experiments. (A) After 5 days of culture (when sporulation is complete), static cultures have produced visible biofilms, while shaken cultures are homogeneous. Note that shaken cultures also contain glass beads to prevent biofilm formation. (B) The relative fitness of rare (10% frequency) pherotypes I and III in competition with pp IV. Boxplots show the median fitness and first and third quartiles, as per Fig. 2.

Before evaluating hypotheses in naturalistic infections, we also wished to test if facultative cheating was robust to an intermediate level of complexity. Here, we manipulated the degree of spatial structure in insect homogenates by competing different pherotypes in shaken and static cultures. We were specifically interested in the impact of increased spatial structure in static cultures on the fitness of potential facultative cheats at low frequency. After cultures had sporulated, there were clear visual differences between static and shaken cultures. Shaken cultures were homogeneous and readily diluted, whereas static cultures formed suspended biofilms resembling pellicles (Fig. 4A). These pellicles needed to be disrupted with repeated aspiration before cultures could be diluted. As predicted, the fitness of rare potential cheats was reduced in static cultures (GLM, *F*_1,93_ = 67.1, *P* < 0.0001) (Fig. 4B). Rare competitors from either pp III or pp I could not, in fact, be described as cheats in static culture since their fitness was always less than the pp IV competitor. Only rare pp III in shaken cultures was able to outcompete its more common competitor. There was a significant interaction between culture treatment and pherotype—treatment effects on pp I were much weaker (GLM, *F*_1,92_ = 12.8, *P* = 0.0006) (Fig. 4B). This is consistent with the higher degree of cross talk between pp I and pp IV QS systems (Fig. 1), which should limit facultative cheating in the shaken cultures.

### Tests of facultative cheating in naturalistic infections.

**FIG 5:**
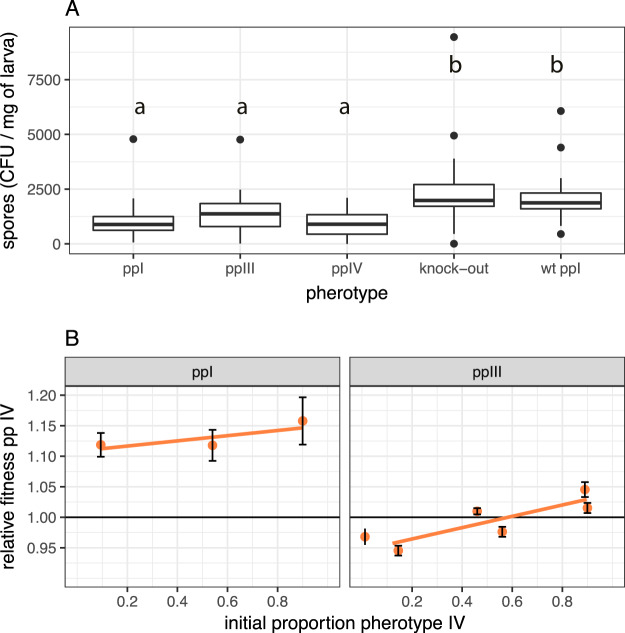
Variation in total reproduction and of relative fitness of naturalistic oral infections of insect larvae. (A) Final population size measured as spore productivity per unit weight of insects for different pherotypes, namely, the QS knockout null mutant Δ*papRplcR* and the QS wild type; boxplots with different letters indicate pherotypes that are significantly different in *post hoc* treatment contrasts. (B) Variation in fitness with frequency of competitors in two sets of competition experiments with different pherotype pairs; data are means ± SE with fitted models for each competition treatment.

In naturalistic oral infections, the main benefit of the PlcR system is in improved infectivity and host invasion from the midgut rather than simple growth. In oral infections, increased signal responsiveness or functioning QS was not expected to increase population size (Fig. 5A). Consistent with this expectation, there was no significant difference in the numbers of spores produced between the Δ*plcRpapR* deletion mutant and the functional WT (Fig. 5A) or between the three pherotypes. This was tested by model simplification; combining deletion mutant and WT treatments and combining all three plasmid-expressed pherotype treatments into a second treatment level resulted in no significant loss of explanatory power (Fig. 5A) (*F_3_*_,158_ = 1.56, *P* = 0.33). The three pherotypes with complemented PlcR/PapR on plasmids did produce fewer spores than the WT (Fig. 5A) (GLM, pherotype main effect, *F*_4,155_ = 11.1, *P < *0.00001), a result consistent with a cost to using an artificial expression system.

Contrary to the facultative cheating hypothesis, natural infections produced positive frequency-dependent fitness in competition experiments (Fig. 5B) (*F*_1,587_ = 23.6, *P < *0.0001). Note that there were clear differences in mean fitness for pp I and pp III in competition with pp IV (Fig. 5B) (*F*_1,587_ = 144.3, *P < *0.0001); pp I strains had much lower fitness in this host, supporting the prediction that important phenotypic differences arise from the carriage of different QS alleles. The magnitude of difference in fitness also corresponds to patterns predicted by responsiveness; the largest difference in fitness occurs between pps I and IV, where we see the greatest difference in signal responsiveness between pherotypes.

In order to validate our use of a plasmid expression system, we also measured the fitness of the pp III and pp IV constructs in competition with the WT (pp I) bacterial background in naturalistic infections. Competition between pp III and pp I WT showed positive frequency-dependent fitness, (see Fig. S1 in the supplemental material) (*F*_1,70_ = 16.2, *P* = 0.00014), while frequency had no effect on the fitness of pp IV in competition with pp I WT (Fig. S1) (*F*_1,90_ = 0.07, *P = *0.79). The relative fitness of the WT pp I strain was increased by ≈10% relative to that of the plasmid-complemented pp I construct (Fig. 3B), an effect consistent with a modest cost of plasmid carriage.

10.1128/mBio.00535-20.1 FIG S1 Competition between plasmid-borne PlcR/PapR constructs and the wild-type group I strain in insects. These experiments qualitatively repeat those in the main body; positive frequency dependence occurred in one of these pairwise competition experiments. Comparisons between the pp IV/pp I experiment here and in the main body suggest that the wild-type chromosomal PlcR/PapR system is slightly more competitive. Data are means ± SE with fitted models for each competition treatment. Download FIG S1, PDF file, 0.2 MB. Copyright © 2020 Zhou et al. 2020 Zhou et al. This content is distributed under the terms of the Creative Commons Attribution 4.0 International license.

### Group size and pherotype fitness *in vivo*.

Since QS is a density-dependent process, pherotypes with quantitative differences in responsiveness to signals might have a relative fitness that varies with group size. Group size in infection is not a constant, but mean group size during infection could vary with either initial dose or the final population size of sporulated bacteria in cadavers. Of these two variables, initial dose had a weaker effect on fitness. In the experiments with pherotypes III and IV that manipulated dose, statistical models with a final population size (Akaike information criterion [AIC] = −929.8; model: fitness ∼ frequency ppIV + sqrt[final spore concentration]) had more explanatory power than models using initial dose (AIC = −926.9; model: fitness ∼ frequency group IV+ log_10_ [dose]). Initial doses also had no significant effect on relative fitness in addition to that of final population size in cadavers (GLM log_10_ [dose], *F*_1,425_ = 0.946, *P = *0.33; dose × frequency interaction, *F*_2,423_ = 2.55, *P* = 0.079). Dose and final population size covaried, so dose could be used as alternative explanatory variable (dose × frequency interaction, *F*_2,426_ = 3.03, *P = *0.049). In these models, pp IV had the greatest competitive fitness at low dose and high frequencies (see Fig. S2 in the supplemental material).

10.1128/mBio.00535-20.2 FIG S2 The effect of initial inoculum dose on the relative fitness of competing pherotypes. Inoculum dose provides an alternative means of assessing density dependent effects on competitive fitness. While the outcome of competition between pherotype III and IV was largely determined by frequencies, dose-dependent effects on fitness were apparent when pherotype III was rare (initial frequency of 10%) and pherotype IV was common (initial frequency of 90%) (dose × frequency interaction, *F*_2,426_ = 3.03, *P *= 0.049). Data are means ± SE with fitted models for each competition treatment. Download FIG S2, PDF file, 0.2 MB. Copyright © 2020 Zhou et al. 2020 Zhou et al. This content is distributed under the terms of the Creative Commons Attribution 4.0 International license.

**FIG 6:**
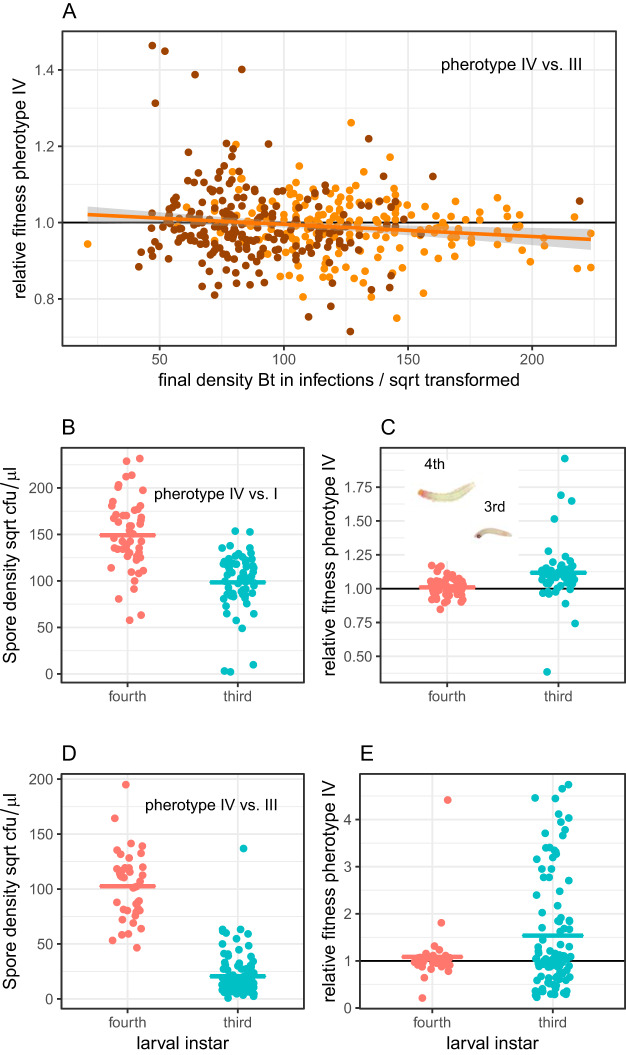
Group size and the relative fitness of competing pherotypes. (A) In competition experiments between group IV and group III, relative fitness covaried with the final population size of B. thuringiensis; data are from competition experiments with 10%, 50%, and 90% pp IV in inocula; colors indicate the two experimental repeats. (B) In competition experiments between pherotypes IV and I, differently sized insect hosts produced strong differences in bacterial group size. (C) The relationship between differences in host size and relative fitness of pp IV and pp I; these experiments used 50% pp IV in inocula; the photograph inset shows relative larval size of fourth and third instar larvae, and the fourth instar larvae is approximately 5 mm long. Competition experiments in different sized insects between pp IV and pp III also produced different final bacterial population sizes (D) and relative fitness (E). Note that in order to display biologically informative zero counts in pairwise competitions, fitness data in E have been transformed by adding minimal countable densities to both genotypes. Where fitness of group IV was >2, no pp III bacteria were detected in cadavers. Data points represent individual hosts; in A, the line represents the fitted linear statistical model, while horizontal bars in B, C, D, and E are means.

Since final population size was more important than dose in determining relative fitness, we explored this variable in more detail. Pherotype IV had higher fitness in infections with lower final densities, after taking into account frequency dependence (Fig. 6A) (GLM square root [density] × frequency interaction, *F*_1,428_ = 3.61, *P* = 0.028; main effect of density, *F*_1,430_ = 8.86, *P* = 0.0031). Independent experimental repeats accounted for quite substantial differences in final pathogen population size (Fig. 6A) (*F*_1,430_ = 177, *P < *0.0001). There are a range of explanations for the correlation between population size and relative fitness. For instance, if pp IV cells invest more in virulence and less in growth, infections dominated by pp IV might produce fewer spores.

However, the simplest explanation for the variation in pathogen population size was that insect host size varied slightly between experimental repeats, and this determined the resources available for bacterial growth. We, therefore, set up additional experiments to explicitly test the relationship between insect host size and pherotype fitness without the potentially confounding influence of variation in dose or frequency. These experiments used 50:50 mixtures of spores and competed pp IV against pherotypes I and III. In the experiment competing pp IV and I, larger hosts produced much larger group sizes in terms of final populations of spores (GLM, main effect of larval instar, *F*_1,110_ = 59.9, *P < *0.00001) (Fig. 6B). This independent experiment also confirmed that smaller insects with lower population sizes increased the relative fitness of the more responsive pp IV (GLM main effect of larval instar, *F*_1,110_ = 13.3, *P* = 0.0004) (Fig. 6C). Experiments competing pp IV and III produced qualitatively similar results; the final population of spores was larger in the larger insects (*F*_1,142_ = 385, *P < *0.0001) (Fig. 6D). The more responsive pp IV had higher mean fitness in the smaller hosts (GLM main effect of instar, *F*_1,142_ = 5.35, *P* = 0.022) (Fig. 6E). However, statistical models were not well behaved for this last experiment, and data were not normally distributed. Nevertheless, an alternative classical test indicated that the pp III pherotype was less efficient at invading smaller hosts, an effect consistent with the known role of QS in facilitating crossing of the midgut barrier (12). In the competitions in smaller third instar insects, the pp III competitor failed to invade hosts significantly more often than pp IV (23 out of 102 cadavers contained no pp III, while only 9 out of 102 cadavers lacked pp IV; Fisher’s exact test, *P* = 0.0114) (Fig. 6E).

## DISCUSSION

We found good support for the hypothesis that different QS pherotypes are maintained by environment-dependent fitness (16). Exchange and replacement of signaling pherotypes had detectable consequences for a range of life history traits, including competitive fitness. Importantly, different QS alleles could increase or decrease the production of virulence factors or the transcription of QS-regulated genes. This phenotypic variability is similar to the variation in timing and expression of QS-dependent virulence factors in Staphylococcus aureus *agr* pherotypes [34). In contrast to work on S. aureus, here, we were able to link phenotypic variation to differences in pathogen fitness and life history *in vivo*. The most ecologically significant result was that more responsive pherotypes were fitter in smaller hosts in naturalistic infections. This makes biological sense for the B. cereus group, as the body sizes of hosts vary over several orders of magnitude (35). One biological interpretation here is that different pherotypes will be favored in differently sized hosts or in ecological contexts with varying signal abundance or persistence.

This study found some support for the hypothesis that facultative cheating can maintain diverse signaling systems in Gram-positive bacteria, although under restricted conditions. Facultative cheating requires particular ecological assumptions, such as the close proximity of colonies exchanging signals by diffusion and intermediate levels of bacterial population structure. The fitness of potential cheats was reduced substantially in static cultures of insect homogenates, which produced visible biofilms, as we have found previously for QS null mutants (12). Shaken liquid cultures are a poor model of *in vivo* infection, while numerous studies have shown that biofilms can increase relatedness via spatial separation of different genotypes and, thereby, limit cheating (39–41). The high levels of clonality in B. thuringiensis and S. aureus infections also make facultative cheating more unlikely since mixed infections must be common enough to drive some negative frequency-dependent fitness (13, 43, 44).

Another important assumption of facultative cheating is that there must be a low level of cross talk between different pherotypes, i.e., weak activation of receptors by signal from different groups. Levels of cross talk between the B. cereus pherotypes corresponded neatly with levels of facultative cheating in shaken insect homogenates, the only environment in which we saw effective cheating. For example, only pp IV could consistently exploit other pherotypes, and pp IV responds very weakly to the signals of the other groups. Pherotype III could exploit pp IV when rare, but rare pp III had a smaller advantage than pp IV in this contest. Cross talk can explain this asymmetry, as pp III responds to pp IV signals more than vice versa. Finally, pp I receptors, which have the highest level of cross-activation by pp IV signals, could not effectively exploit pp IV when rare at all (i.e., fitness was always <1.0). While the environment-dependent and facultative cheating hypotheses for the maintenance of polymorphic QS are not mutually exclusive, the requirements for facultative cheating—intermediate relatedness, no spatial structure, and no cross talk—make it unlikely to be a major force in the evolution of the B. cereus group.

Facultative cheating by weak responders also rests on the assumption that cooperation (the shared fitness benefits of QS signals and QS-regulated products) is prevalent in the first place. While the insect homogenate setup shows the hallmarks of a classic public goods systems (effective cheating and shared benefits in terms of group level reproduction), natural infections do not (12). As we have shown previously, there is limited evidence for cooperative QS in naturalistic infections, unlike in insect homogenates (12). Insect homogenates and naturalistic oral infections also showed very different patterns of bacterial reproduction and frequency-dependent fitness in this study. To understand these differences, it is important to spell out how naturalistic infections of B. thuringiensis differ from standard QS theory. The fundamental mechanism of QS systems means that the expression of quorum-regulated traits should increase with local population size or local signal abundance (2, 14). The most successful explanation of this density dependence is based on social evolution theory (9, 10). In these models, the costs of traits are borne by individual pathogens, while the benefits are shared within groups, and as group size increases, so should the total benefits to infecting pathogens (9, 10). In this framework, the selected optimal expression of nonobligate virulence factors increases with group size. This is because the benefits of expressing virulence factors in larger groups eventually offset the individual cost of investment (9, 10).

This density dependence only emerges for nonobligate virulence factors (9, 10), and so, it is important to consider whether the PlcR QS system is obligately required for reproduction in B. thuringiensis. In live hosts, the lytic enzymes of the PlcR regulon do not have a simple nutritional role as they do in insect homogenates; instead, these enzymes assist in the breakdown of the gut lining and allow pathogens to access resources in the body of the host (26). The situation is complicated by the fact that in B. thuringiensis, the characteristic Cry toxins that define this species play a more dominant role in infection and also breakdown the midgut epithelium (45). Nevertheless, in some hosts, such as *Galleria melonella*, loss of the PlcR-based QS system has a dramatic impact on mortality and infection (26, 29). The host used in this study, *P. xylostella*, is highly sensitive to Cry toxins and PlcR null mutants can invade this insect successfully, indicating that this QS system is not obligate for every host species. Nevertheless, QS null mutants have a reduced ability to invade the hemocoel of *P. xylostella* from the gut (12). Thus, if PlcR is not always strictly obligate, it does have a very large impact on infection success in multiple host species. Note that these benefits are clearer at modest doses of Cry toxin and at lower spore doses (12, 26, 29). At high Cry doses, the insect midgut disintegrates and readily allows entry of microbes—this could explain why our double knockout mutant reproduced effectively here; we used a dose of Cry toxin that guaranteed mortality.

In addition, do the products regulated by PlcR provide group-level benefits that can be exploited by nonproducers or potential cheats? Nonproducers cannot outcompete functional PlcR strains in oral infections (12). This study also shows that lower expressing variants do not consistently outcompete higher expressing variants, which is another key prediction of social evolution. For spatially structured infections, we might also expect negative frequency dependent fitness since rare nonproducers should be better able to access benefits when producers are common (46). While we have seen this pattern in competition experiments with nonproducers (12), this was not repeated for the rare pherotypes in this study. Additionally, in Gram-positive species, QS null mutants are rare in natural populations (12, 47, 48), supporting experimental data showing that QS nonproducers have low fitness. In summary, there may be some shared benefits to QS, but these indirect fitness benefits are relatively weak.

The positive frequency-dependent fitness of competing pherotypes in this study was unexpected. However, this result is consistent with previous work which manipulated the dose and frequency of QS and QS null mutants in PlcR and showed that the total abundance of wild-type bacteria predicted the probability of successful infections (12). It appears that the total availability of QS signal in B. thuringiensis infections can determine infection success and fitness, especially at lower doses. Common pherotypes may have higher fitness because their cognate signal is more abundant *in vivo*. This argument holds if QS signals are more readily shared than the products activated by QS. Signals do confer a greater shared benefit than the products of QS regulated by PlcR (12), and this makes biological sense given that peptide signals are small and diffusible, while half of the PlcR-regulated enzymes are bound to the cell wall (25). Previous work also provides some evidence for the existence of spatially separated microcolonies in the gut in the early stages of infection, when PlcR is active (12). Potentially, then, different microcolonies with the same pherotype can increase QS-regulated expression in response to each other within the gut. Statistical simulations of competition between B. thuringiensis genotypes suggest that competition manifests as a race to cross the midgut and enter the resource-rich body of the insect (43). Since QS-regulated products are required to cross the midgut barrier, small differences in the speed of activation of PlcR could be important in this race. This study also showed that less-responsive pherotypes (pp III and I) could have reduced invasion relative to more responsive variants at a low group size. This also fits with a model in which signal abundance is limiting when group size is low.

Predictions relating to group size during infection were challenging to test since it is hard to manipulate the number of bacteria within an ongoing infection and even harder to be confident that this manipulation occurs when quorum sensing is happening. Group size in early infection might be affected by ingested dose, but here, ingested dose had very weak effects on fitness. This is potentially because dose has only a transient impact on group size, which occurs before spores have germinated (12), and before PlcR/PapR-dependent QS can begin. In contrast, host size and final pathogen density had robust impacts on the fitness of QS variants. Simply based on the size of the gut, smaller hosts should contain a reduced number of newly germinated spores, in addition to placing a limit on the final population size. With the above caveats in mind, the observation that more productive genotypes are fitter in smaller groups contrasts with the predominant QS theory (9, 10).

In fact, the observation that optimal expression declines with group size corresponds to the predictions for obligate virulence factors and the “many hands make light work” analogy used in key theoretical analyses of QS (9). In other words, optimal investment in virulence should decline with group size if a fixed amount of virulence factors are required for infection (9). Other explanations for low group sizes favoring high expression are possible. For instance, the transit times of ingested material are shorter in the guts of smaller hosts. Earlier insect instars also molt and shed their gut lining more frequently, so more responsive pherotypes may be fitter in smaller hosts because they can increase the production of virulence factors more rapidly and invade hosts before they are expelled.

If PlcR does not conform to the public goods model of QS, how do we explain the regulatory structure shaping density-dependent expression of virulence? We hypothesize that PlcR/PapR primarily acts by sensing microcolony formation and the spatial distribution of bacteria after attachment to the midgut (49); the mean population size in the host as a whole may be much less important. It may be critical that PlcR is an autoregulator—activation of *plcR* increases the production of the PlcR receptor protein and the papR signal (50)—so, QS responses have positive feedbacks which produce very rapid activation (29). This may have implications for differences between pherotypes; potentially more responsive variants may be able to accelerate expression more quickly during the vital early phase of infection.

In previous evolutionary studies of QS, signaling and QS-regulated traits are public goods, with strong indirect fitness benefits and high fitness at low frequencies (4, 7, 9, 10, 51). Importantly, for Gram-negative models, cooperation has been seen *in vitro* and *in vivo* (7). In this study, public goods cooperation occurred only in insect homogenates. Overall, PlcR does not conform well to a public goods model in insect infections; group-level benefits are very weak, and the variation in fitness with group size corresponds to predictions for obligate virulence factors, rather than for typical QS traits. We, therefore, need an alternative explanation for the density-dependent regulation of virulence for PlcR. Social evolution models cannot also easily explain the observed polymorphism in peptide signals. A model of environment-dependent fitness for QS variants seems more relevant for some of the best-studied Gram-positive pathogens, namely, S. aureus and the B. cereus group, since different allelic variants can show differences in gene expression, fitness, life history, and potentially distinct host niches (52, 53).

## MATERIALS AND METHODS

### Bacterial strains.

Distinct *plcR-papR* genes were sourced from three of the four pherotype groups found in B. cereus *sensu lato.* Pherotype IV, III, and I genes were derived from B. thuringiensis serovar roskildiensis, B. thuringiensis serovar kurstaki, and B. thuringiensis subsp. *berliner*, respectively, and introduced on the stable pHT304 plasmid (54) into the acrystalliferous strain B. thuringiensis 407 Cry^−^ A’Z Δ*plcR-papR* which contains a *lacZ* fusion for reporting *plcR* transcriptional activity (23). Experiments also used the 407 Cry^−^ A’Z Δ*plcRpapR* mutant lacking the complemented plasmid, as well as the wild-type B. thuringiensis 407 Cry^−^ isolate with the intact chromosomal *plcR-papR* genes, which was the source of the pherotype pp I gene, produced as described previously (29). In order to distinguish between pherotypes, the pp IV plasmid strain was constructed to be tetracycline resistant, while the pp I and pp III plasmids retained the erythromycin-resistance gene as a marker. Details of cloning procedures are fully described in Text S1 in the supplemental material.

10.1128/mBio.00535-20.3 TEXT S1 Supplementary methods. Download Text S1, DOCX file, 0.1 MB. Copyright © 2020 Zhou et al. 2020 Zhou et al. This content is distributed under the terms of the Creative Commons Attribution 4.0 International license.

### PlcR activity assays.

Two assays were used to compare the expression of genes under the control of the PlcR QS system under standard laboratory conditions. The first assay assessed the hydrolysis of egg yolk phosphatidylcholine by the lecithinase enzyme PlcB (55), whose encoding gene is a member of the PlcR regulon in B. cereus (25). In the second set of assays, we measured the transcription of the *plcA* gene, also under the control of PlcR (50), using a transcriptional fusion between the promoter of this gene and the reporter gene *lacZ* encoding a β-galactosidase enzyme. Specific activity in the β-galactosidase and lecithinase assays was as described previously (55) and repeated five and four times, respectively. Lecithinase activity was measured in the filtered supernatant of cells from liquid culture and was calculated as the slope of the linear part of the curve/[optical density at 600 nm (OD_600_) of the culture at the time of sampling × volume of supernatant used in the assay (liters)], while β-galactosidase was measured in disrupted cells from liquid culture and was calculated according to (OD_420_ × 1,500,000)/(reaction time in min × vol crude extract in μl × *C*) where *C* is the protein concentration.

### Infection experiments.

Competition experiments using oral infection of diamondback moth Plutella xylostella larvae or culture in homogenized insect medium followed published protocols (12), except that insect homogenates were not pasteurized before maceration. Experiments with homogenates used shaken cultures in 24-well plates containing two sterile glass beads rotated at 180 rpm or static cultures without glass beads. All larvae were reared aseptically on sterile diet after being hatched from surface-sterilized eggs (56). Competition experiments based on oral infections of pp III and pp IV used a full factorial design with 5 frequencies of competing strains (approximately 100%, 90%, 50%, 10%, and 0% of group IV) and three doses (2 × 10^3^, 1 × 10^4^, and 5 × 10^4^ spores μl^−1^), with 25 insects (third instar) in each treatment. These experiments were repeated twice. Competition experiments using pp I used three frequencies (90%, 50%, and 10% of group I) and a dose of 1 × 10^4^ spores ^−1^ and 30 to 35 larvae per treatment. Invasion experiments with static and shaken insect homogenates used cultures containing 90% pp IV and 10% pp I or pp III. Competition experiments in hosts of different sizes used third and fourth instar *P. xylostella* larvae with 50:50 mixtures of pp I and IV and a 50:50 mix of pp III and IV.

### Data analysis.

Measurements of fitness were based on reproductive rates for competing genotypes based on viable spores in inocula and in cadavers/or media after growth was complete. The density and relative frequency of competing genotypes were calculated by culturing spore preparation on selective media containing erythromycin or tetracycline (10 μg ml^−1^). Scoring of initial ratios used a minimum of 800 colonies. Bacterial relative fitness (*W*) was calculated using the ratio of Malthusian parameters or relative growth rate (*W_A_*_,_*_B_ = log_e_* [final^A^/initial^A^]/*log_e_* [final^B^/initial^B^]) where final^A^ and final^B^ refer to the final and initial numbers of strains A and B in culture or in an infection (57). The initial numbers of cells initiating a live infection are hard to calculate directly since the vast majority of ingested spores are excreted before germinating *in vivo* (12). Nevertheless, microscopic examination of sections of insects suggest a lower bound of 10 cells for this bottleneck (12). We estimated the infection bottleneck at 50 cells, a conservatively high estimate based on previous experiments (43). Nevertheless, sensitivity analyses have shown that calculations of fitness are insensitive to this parameter (see Fig. S3 in the supplemental material). It is not possible to calculate relative fitness when there is no detectable growth in hosts from either competitive partner. In the pp III and IV competition experiments with differently sized larvae, zero cell counts were transformed by the addition of the minimal detectable value, principally to allow visualization of results. Other data were log_10_- or square root-transformed to improve homoscedasticity as required. Relative fitness and bacterial reproduction were analyzed using GLMs; peak enzyme activity data were analyzed in mixed linear effect models with experimental replicate as a random factor. Planned *post hoc* comparisons between treatment levels in GLMs were conducted using model simplification and pooling of treatment levels. Thus, if combining treatment levels resulted in significant loss of deviance, treatments were judged to have different effects. Where error structures or data failed to meet tests of normality or homoscedasticity (produced by zero counts) data were analyzed with classical tests (Fisher’s exact test). All statistical analyses were performed in R v3.3.2 (R Core Team 2013).

10.1128/mBio.00535-20.4 FIG S3 Sensitivity analysis on infection bottlenecks and estimation of this parameter affect the calculation of relative fitness in competition experiments. The data used in fitness calculations are the same as those used in Fig. 5A, which was based on a bottleneck of 50 cells. Estimation of bottleneck size is used to estimate bacterial reproduction *in vivo* (Malthusian parameters), i.e., log_2_(final density/initial density), where initial density is the infection bottleneck multiplied by the proportion of given genotype in inocula. Download FIG S3, PDF file, 0.1 MB. Copyright © 2020 Zhou et al. 2020 Zhou et al. This content is distributed under the terms of the Creative Commons Attribution 4.0 International license.

### Data availability.

Experimental data on expression, bacterial densities, and relative fitness supporting this publication are openly available from the University of Exeter’s institutional repository at https://doi.org/10.24378/exe.1843. 
