# Supplementary material for: Optimal Response to Quorum-Sensing Signals Varies in Different Host Environments with Different Pathogen Group Size
Source: mBio. 2020 Jun 2;11(3):e00535-20. doi: 10.1128/mBio.00535-20 (PMC7267880; doi:10.1128/mBio.00535-20)

Supplementary Figure S1. Competition between plasmid borne plcR/papR constructs and the wild type Group I strain in insects. These experiments qualitatively repeat those in the main body: positive frequency dependent occurred in one of these pairwise competition experiments. Comparisons between the pp IV / pp I experiment here and in the main body suggests that the wild type chromosomal PlcR / PapR system is slightly more competitive. Data are means ± SE with fitted models for each competition treatment.

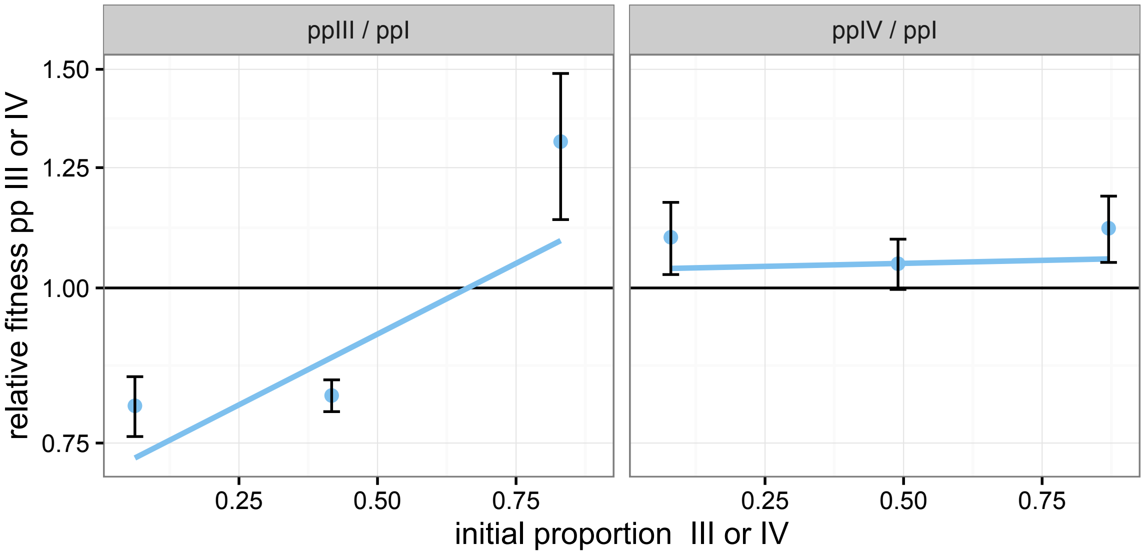

Supplement: FIG S1 [file mBio.00535-20-sf001.pdf]
