# Supplementary material for: Optimal Response to Quorum-Sensing Signals Varies in Different Host Environments with Different Pathogen Group Size
Source: mBio. 2020 Jun 2;11(3):e00535-20. doi: 10.1128/mBio.00535-20 (PMC7267880; doi:10.1128/mBio.00535-20)

Supplementary Figure S2. The effect of initial inoculum dose on the relative fitness of competing pherotypes. Inoculum dose provides an alternative means of assessing density depends effects on competitive fitness. While the outcome of competition between pherotype III and IV was largely determined by frequencies, dose dependent effects on fitness were apparent when pherotype III were rare (initial frequency of 10%) and pherotype IV common (initial frequency of 90%) (dose * frequency interaction $F_{2,426}$ = 3.03, P = 0.049). Data are means ± SE with fitted models for each competition treatment

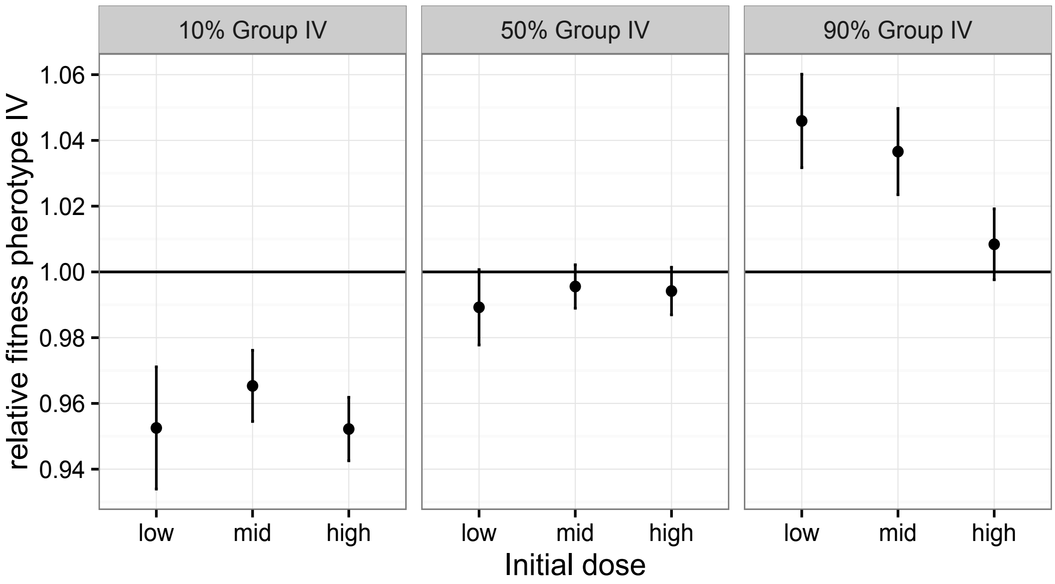

Supplement: FIG S2 [file mBio.00535-20-sf002.pdf]
