# Supplementary material for: Optimal Response to Quorum-Sensing Signals Varies in Different Host Environments with Different Pathogen Group Size
Source: mBio. 2020 Jun 2;11(3):e00535-20. doi: 10.1128/mBio.00535-20 (PMC7267880; doi:10.1128/mBio.00535-20)
Supplement: TEXT S1 [file mBio.00535-20-s0001.docx]

Supplementary Materials and Methods for “Optimal response to quorum-sensing signals varies in different host environments with different pathogen group size”

Bacterial strains and growth conditions

The acrystalliferous *Bacillus thuringiensis* 407 Cry^-^ strain (Bt 407^-^) (Lereclus *et al.*, 1989), the *B. thuringiensis* subsp. *kurstaki* HD-73 and the *B. thuringiensis* serovar *roskildiensis* strains (from the INRA collection)*.* Bt 407^-^ A’Z *ΔplcR-papR* (Bouillaut et al 2008) was used as the parental strain to create the strains described below. *E. coli* strain DH5α (Taylor *et al.*, 1993) was used as the host strain for plasmid construction. *E. coli* strain ET12567 (MacNeil *et al.*, 1992) was used to prepare DNA prior to electroporation in *B. thuringiensis*. Unless otherwise noted, cells were grown in LB medium (1% tryptone, 0.5% yeast extract, 1% NaCl) at 37°C and stored at -80°C in LB containing 15% glycerol.

The antibiotic concentrations used for selection of *B. thuringiensis* and *E. coli* were as follows: erythromycin, 10µg/mL; tetracycline, 10µg/mL; kanamycin, 200µg/mL; ampicillin, 100µg/mL.

DNA manipulation

Chromosomal DNA was extracted from *B. thuringiensis* cells using the Puregene DNA Purification Kit (QIAgen, France). Plasmid DNA was extracted from *E. coli* using QIAprep spin columns (QIAgen, France). Restriction enzymes and T4 DNA ligase (New England Biolabs, USA) were used in accordance with the manufacturer’s recommendations. Oligonucleotide primers were synthesized by Sigma-Proligo (Paris, France). PCRs were performed in a 2720 Thermak cycler (Applied Biosystems, USA) using the Phusion^®^ High-Fidelity DNA Polymerase or the Standard Taq DNA polymerase (New England Biolabs). Amplified fragments were purified using the QIAquick PCR purification Kit (QIAgen). Digested DNA fragments were separated on 0.8% agarose gels after digestion and extracted from gels using the QIAquick gel extraction Kit (QIAgen). All the constructs were verified by sequencing (Beckman Coulter Genomics, Takeley, UK).

Plasmid and strain construction

Plasmid pHT304 (Arantes and Lereclus 1991) that confers resistance to erythromycin was modified as follows to confer resistance to tetracycline instead. Plasmid pHT304 was amplified, except for the gene conferring resistance to erythromycin, using primers F-pHT315 and R-pHT315 (5-3’ TCCCCGCGGCGTTACTAAAGGGAATGG and CCGCTCGAGCTTAATTACAAATTTTTAGC respectively) and digested with *Sac*II and *Xho*I. The gene conferring resistance to tetracycline was amplified from plasmid pHT1618 (Lereclus and Arantes 1992) using primer pair F-tet/R-tet (5-3’ CCGCTCGAGCCATATTGTTGTATAAGTGATG and TCCCCGCGGCAATAACCCTGATAAATGCTTC respectively) and cloned between the *Sac*II and *Xho*I restriction sites of the newly amplified pHT304 fragment, creating plasmid pHT304-tetR.

Plasmid pHT304-plcRpapR_III_ was constructed by inserting the plcR-papR genes, amplified using primers F-S1X and R-S2H (5-3’ GCTCTAGACTATTATTATATGTGAGATGAATTGTATG and CCCAAGCTTGTAAAGACGTTTGGATGTTACTCC respectively) and the genomic DNA from strain *B. thuringiensis* subsp. *kurstaki* HD-73 as a template, between the *Xba*I and *Hind*III restriction sites of plasmid pHT304. The plasmid was then transformed into Bt 407^-^ A’Z *ΔplcR-papR* cells. The resulting Bt 407^-^ A’Z *ΔplcR-papR* *(*pHT304-plcRpapR_III_) strain was designated Group III strain throughout the manuscript.

Plasmid pHT304-tetR-plcRpapR_IV_ was constructed by inserting the plcR-papR genes, amplified using primers F-S1X and R-S2LZ (5-3’ ACATGCATGCCTAGTAAAGACGTTTGGATGTTACTCC) and the genomic DNA from strain *B. thuringiensis* serovar *roskildiensis* as a template, between the *Xba*I and *Sph*I restriction sites of plasmid pHT304-tetR. The plasmid was then transformed into Bt 407^-^ A’Z *ΔplcR-papR* cells. The resulting Bt 407^-^ A’Z *ΔplcR-papR* *(*pHT304-tetR-plcRpapR_IV_) strain was designated pp IV strain throughout the manuscript.

**References**

Arantes O, Lereclus D (1991). Construction of cloning vectors for *Bacillus thuringiensis*. *Gene* **108:** 115-119.

Bouillaut L, Perchat S, Arold S, Zorrilla S, Slamti L, Henry C *et al* (2008). Molecular basis for group-specific activation of the virulence regulator PlcR by PapR heptapeptides. *Nucleic acids research* **36:** 3791-3801.

Lereclus, D., Arantes, O., Chaufaux, J., and Lecadet, M. (1989). Transformation and expression of a cloned delta-endotoxin gene in *Bacillus thuringiensis*. *FEMS Microbiol Lett* 51**,** 211-217.

Lereclus D, Arantes O (1992). spbA locus ensures the segregational stability of pTH1030, a novel type of Gram-positive replicon. *Molecular Microbiology* **6:** 35-46.

Macneil, D.J., Gewain, K.M., Ruby, C.L., Dezeny, G., Gibbons, P.H., and Macneil, T. (1992). Analysis of *Streptomyces avermitilis* genes required for avermectin biosynthesis utilizing a novel integration vector. *Gene* 111**,** 61-68.

Slamti L, Lereclus D (2005). Specificity and polymorphism of the PlcR-PapR quorum-sensing system in the *Bacillus cereus* group. *Journal of Bacteriology* **187:** 1182-1187.

Taylor, R.G., Walker, D.C., and Mcinnes, R.R. (1993). *E. coli* host strains significantly affect the quality of small scale plasmid DNA preparations used for sequencing. *Nucleic Acids Res* 21**,** 1677-1678.
