# Supplementary material for: Optimal Response to Quorum-Sensing Signals Varies in Different Host Environments with Different Pathogen Group Size
Source: mBio. 2020 Jun 2;11(3):e00535-20. doi: 10.1128/mBio.00535-20 (PMC7267880; doi:10.1128/mBio.00535-20)

Supplementary Figure S3. Sensitivity analysis on infection bottlenecks and estimation of this parameter affects calculation of relative fitness in competition experiments. The data used in fitness calculations are the same those used in figure 5A, which was based on a bottleneck of 50 cells. Estimation of bottleneck size is used to estimate bacterial reproduction *in vivo* (Malthusian parameters) i.e. $\log_2$(final density/initial density), where initial density is the infection bottleneck multiplied by the proportion of given genotype in inocula.

Bottleneck 10 cells

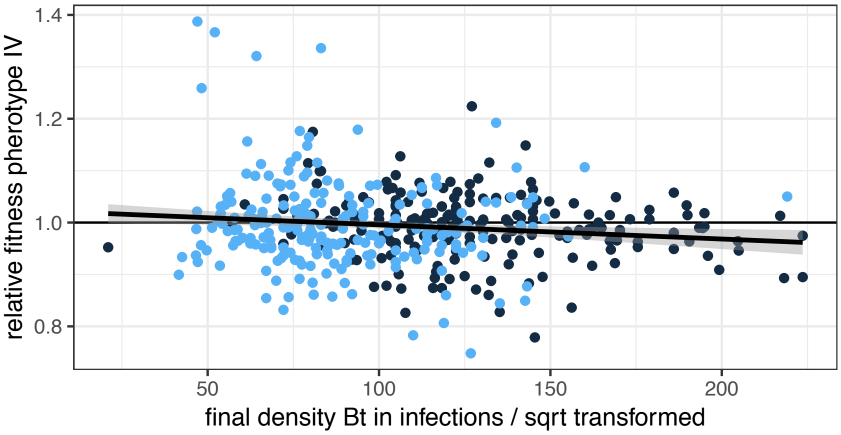

Bottleneck 50 cells

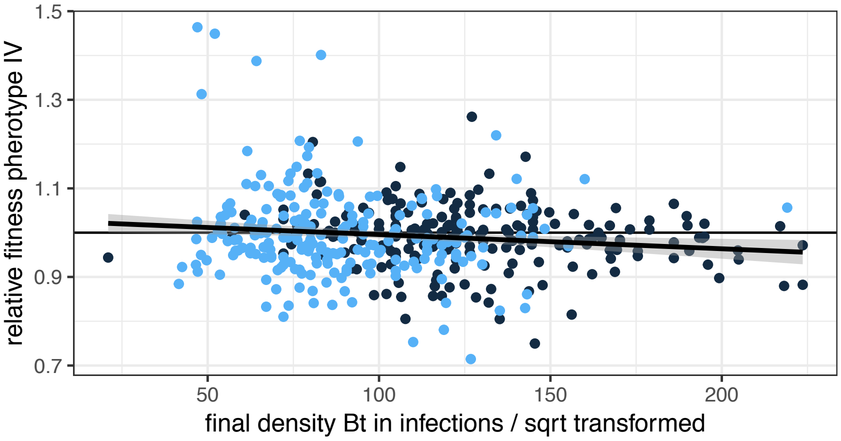

Bottleneck 250 cells

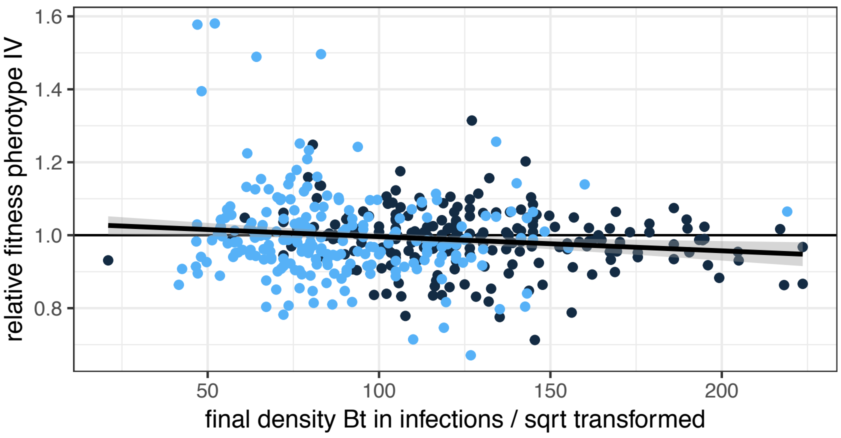

Supplement: FIG S3 [file mBio.00535-20-sf003.pdf]
